# Epidemiologic Features of Traumatic Fractures in Children and Adolescents: A 9-Year Retrospective Study

**DOI:** 10.1155/2019/8019063

**Published:** 2019-02-20

**Authors:** Hongwei Wang, Chencheng Feng, Huan Liu, Jun Liu, Lan Ou, Hailong Yu, Liangbi Xiang

**Affiliations:** ^1^Department of Orthopedics, General Hospital of Northern Theater Command of Chinese PLA, Shenyang, Liaoning 110016, China; ^2^State Key Laboratory of Robotics, Shenyang Institute of Automation, Chinese Academy of Science, Shenyang, Liaoning 110016, China; ^3^State Key Laboratory of Materials Processing and Die & Mould Technology, Huazhong University of Science and Technology, Wuhan, Hubei 430074, China; ^4^State Key Laboratory of Trauma, Burn and Combined Injury, Third Military Medical University, Chongqing 400038, China; ^5^Department of Orthopedics, Xinqiao Hospital, The Third Military Medical University, Chongqing 400037, China; ^6^Department of Orthopedics, Affiliated Traditional Chinese Medicine Hospital, Southwest Medical University, Luzhou 646000, China; ^7^Department of Radiology, Southwest Hospital, The Third Military Medical University, Chongqing 400038, China

## Abstract

**Purpose:**

Fractures are common among all types of paediatric injuries, with differences in incidence over time. Here, we present the epidemiologic features of traumatic fractures in a population of youth ≤ 18 years of age who were admitted to our university-affiliated hospitals from 2002 to 2010.

**Methods:**

We retrospectively reviewed 2450 children and adolescents who had traumatic fractures. The data include variables such as age, sex, date of injury, and the mechanism of injury. For the period of 2002-2010, there were 2450 injury events that resulted in at least 1 fracture.

**Results:**

Low falls (1042, 42.5%) and upper limb fractures (1068, 43.6%) were the most common aetiologies and fracture sites. With increasing age, the proportion of injuries due to motor vehicle collisions (MVCs) decreased and the injuries due to being hit by others and due to sprains increased. With increasing age, the proportion of craniofacial fractures (CFFs) decreased, and lower limb fractures (LLFs), spinal fractures (SFs), and fractures of ribs and the sternum (RSFs) increased. Over time, the proportion of injuries due to MVCs and mechanical injury decreased. Male patients presented with a significantly higher proportion of injuries due to low falls, being struck by an object, being hit by others, and due to sprains. There were a significantly lower proportion of injuries due to MVCs in female patients. Male patients presented with a significantly higher proportion of ULFs in the ≤6-year-old age group and a significantly lower proportion of LLFs and SFs in the 12-18-year-old age group than did female patients.

**Conclusions:**

Low falls and upper limb fractures were the leading cause and fracture sites. To further improve the prevention and treatment of traumatic fractures in children and adolescents, policy makers should pay attention to these characteristics.

## 1. Introduction

Fractures in children and adolescents (≤ 18 years of age) accounted for more than 15% of all traumatic fractures among all age groups in China [1]. Fractures comprise 10% to 25% of all types of paediatric injuries [2–4], and the incidence of fractures increased over time [5, 6]. The patterns of fractures vary between countries, which is due to different climate, culture, and leisure-time activities [7–18].

Paediatric fracture incidence rates have different patterns in different countries. The incidence of girls presenting with a fracture decreased from 1993-1994 to 2005-2006 in Malmö [13]. The childhood fracture incidence decreased significantly during the last two decades in Helsinki, Finland. Concurrently, the incidence of forearm and upper arm fractures increased by one-third [17]. Clavicle and distal radius fractures showed significant seasonal variation for all age groups but not for femoral shaft fractures [18]. In recent years, developments of transportation and construction industry have happened in China and traffic laws were instituted after May 1, 2004. However, data on the characteristics of traumatic fractures (TFs) including age, sex, year of admission, and specific characteristics are scarce in China. In the present study, we present the epidemiologic features of TFs in a population of youth ≤ 18 years of age, who were admitted to our university-affiliated hospitals from 2002 to 2010.

## 2. Materials and Methods

### 2.1. Study Population

We retrospectively reviewed 2450 children and adolescents who had TFs. This study was a retrospective cross-sectional study using data from the Military Hospital Information Registry Database gathered from January 2002 to December 2010. Data were collected from the 2 largest tertiary hospitals located in the Shapingba district, which is a core district located in the northwest of Chongqing city. The data include variables such as age, sex, date of injury, and the mechanism of injury. We also describe age- and sex-related differences in incidence, in the mechanism of injury, and in fracture location. We made definitive diagnoses of TFs in patients who were children or adolescents (≤18 years old) using X-rays, computed tomography (CT), and magnetic resonance imaging (MRI). The aetiologies included motor vehicle collisions (MVCs), high fall (height ≥2 m), low fall (height <2 m), being struck by an object, hit by others, sprain, mechanical injury (such as twist/strikes/cutting injury by machine), and others (such as earthquake injury and firecracker blast injury). Fracture sites included upper limb fractures (ULFs), lower limb fractures (LLFs), craniofacial fractures (CFFs), spinal fractures (SFs), and fractures of ribs and the sternum (RSFs). Informed consent was obtained from the legal guardians when patients came to our hospital, and they were asked whether we could use the data for research. The study protocol and this manuscript were approved by the committee on ethics and the institutional review board of Xinqiao Hospital, the Third Military Medical University. We confirm that all methods were performed in accordance with the relevant guidelines and regulations.

### 2.2. Statistical Analysis

All statistical analyses were performed using SPSS version 22.0 (SPSS, Inc., Chicago, IL). We used Pearson chi-squared tests to assess differences in age, the sex distribution and clinical characteristics between the two groups. Differences in the continuous variables between the two groups were evaluated using independent sample t-tests. The data was normally distributed and was expressed as the mean ± standard deviation (SD).

## 3. Results

### 3.1. Age, Gender, and Year of Admission Distributions

**Figure 1:**
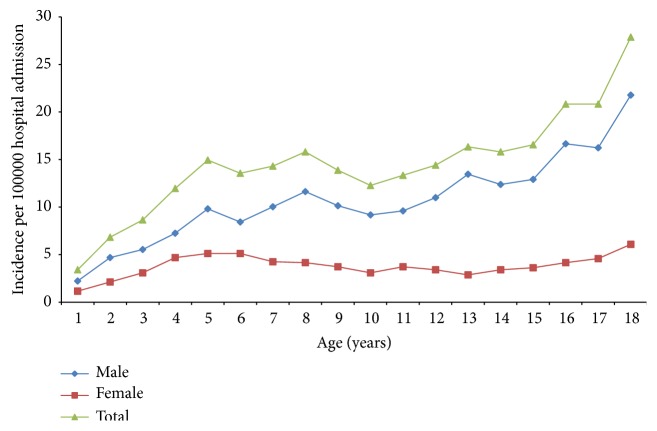
Age- and sex-specific incidence of fractures per 100,000 hospital admissions.

The study enrolled 2450 patients (1808 male and 642 female) with a mean age of 11.1±5.0 years and a sex ratio of 2.8 (Table 1). The mean age of the male patients was significantly higher than the female patients. The number of children diagnosed increased with the year of admission (Figure 1). During all periods the occurrence increased with increasing age groups and a male preponderance was observed in all age groups (Table 2). Female patients presented with significantly higher proportions in the ≤6-year-old age group and significantly lower proportions in the 12-18-year-old age group than male patients (Table 3). The overall annual incidence of TFs was (254.1±62.8) cases per 100000 hospital admissions per 3-year period. Annual incidence rates increased from 186.1 cases (2002-2004) to 309.7 cases (2005-2007) and then decreased to 266.6 cases (2008-2010) with year of admission per 100000 hospital admissions per 3-year period. The incidences had a month and season variation, with peaks in summer (27.2%) and September (9.8%). A significantly lower incidence of TFs was observed in winter (21.5%) than the other three seasons.

### 3.2. Aetiology

Overall, the most common aetiologies were low falls (n=1042, 42.5%), followed by MVCs (n=728, 29.7%) and high falls (n=350, 14.3%) (Table 1). The frequency of low falls was significantly higher in children aged 6-12 years than the other two age groups. The frequency of MVCs was significantly higher in children aged ≤6 years than the other two age groups. The frequency of hit by others was significantly higher in children aged 12-18 years than the other two age groups. With increasing age, the proportion of injuries due to MVCs decreased and injuries due to being hit by others and sprains increased. With increasing year of admission, the proportion of injuries due to MVCs and mechanical injury decreased. Male patients presented with a significantly higher proportion of injuries due to low fall, being struck by an object, being hit by others, sprains, and a significantly lower proportion of injuries due to MVCs than female patients (Table 3).

### 3.3. Fracture Sites

**Figure 2:**
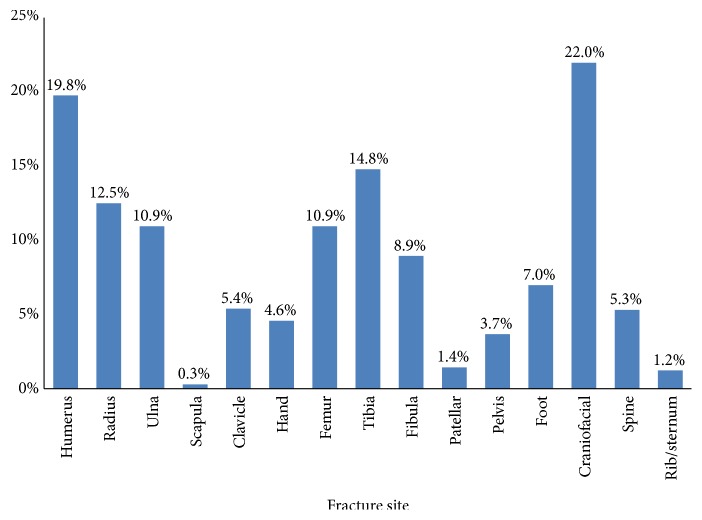
Frequency of specific fracture sites among all the patients.

Among all the patients, the most common fracture sites were ULFs (n=1068, 43.6%) and LLFs (n=925, 37.8%), followed by CFFs (n=538, 22.0%) (Table 1; Figure 2). The proportion of ULF was the largest in the mechanical injury group (83.3%), low fall group (67.0%), and lowest in the MVCs group (17.2%). The proportion of LLFs was the largest in the sprain group (75.5%) and MVCs group (59.8%) and lowest in the hit by other groups (9.5%). The proportion of CFFs was the largest in the hit by others group (54.1%) and lowest in the sprain group (0) and low fall group (9.5%). The proportion of SF was the largest in the high fall group (16.3%).

The most common fracture sites were ULFs (n=209, 37.6%) in the ≤6 year old patients, ULFs (n=403, 51.2%) in the 6-12 year old patients, and LLFs (n=472, 42.6%) in the 12-18 year old patients. The frequency of ULFs was significantly higher in children aged 6-12 years than the other two age groups. The frequency of LLFs was significantly higher in children aged 12-18 years than the other two age groups. The frequency of CFFs was significantly higher in children aged ≤6 years than the other two age groups. The frequency of SFs was significantly higher in children aged 12-18 years than the other two age groups. With increasing age, the proportion decreased in CFFs and increased in injuries due to LLFs, SFs, and RSF. Male patients presented with a significantly higher proportion of ULFs and significantly lower proportion of LLFs and SFs than female patients (Table 3).

### 3.4. Neurological Deficit (ND), Complications, and Associated Injuries (ASOIs)

**Figure 3:**
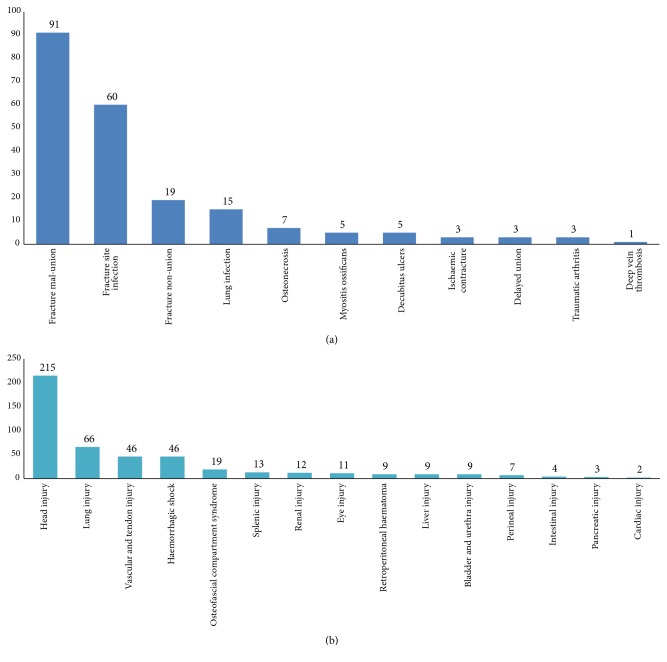
Number of patients presented with associated injuries (a) and complications (b).

A total of 463 (18.9%) patients suffered a neurological deficit (ND), 198 (8.1%) patients had sustained complications (Figure 3(a)), and 385 (15.7%) patients sustained ASOIs (Figure 3(b)). The frequency of ND increased with age (Table 2).

## 4. Discussion

In the current study, fractures were more common in males than females and the sex ratio was 2.8. The result was consistent with previous studies [5, 7, 14]. The peak incidence occurred in the 12-18-year-old age group for both males and females. Male patients presented with a significantly higher proportion of the ≤6-year-old age group and significantly lower proportion of the 12-18-year-old age group than female patients. In previous studies, the peak incidence occurred in different age groups between males and females and all the results were similar [5, 7, 14, 17]. In Sweden, the peak incidence occurred in 13-14 years in boys and in 11-12 years in girls [5]. Fracture incidence had a peak incidence at 14 years of age among boys and 11 years of age among girls in Britain [7]. In Helsinki, the fracture incidence peaked at 14 years in boys and 10 years in girls [17]. There may be a little bias because these studies focus on fractures including a small number of nontraumatic fractures rather than only traumatic fracture in our study.

The leading causes were low falls, which was consistent with previous studies [5, 17]. In Britain, an increase in fracture incidence was seen from 1967 to 1983, but a significant decrease was seen from 1983 to 2005 [17]. In the current study, the annual incidence of TFs increased from 186.1 to 309.7 cases during the first several years. The phenomenon can be explained by the rapidly changing society due to transportation development and the construction industry. The annual incidence of TFs then decreased during the last several years because traffic laws were instituted after May 1, 2004. The level of education and establishment of traffic laws may contribute to declining rates of injuries and fractures. Therefore, we can see that improved traffic safety as well as increased supervision of physical activities also plays an important role in the decreased incidence.

The most common fracture regions were ULF and LLF. The craniofacial fracture and humerus fracture were the most common fracture sites. In previous studies, the most common fracture site was forearm fractures [5, 7, 17, 18]. With increasing age, the proportion of injuries due to MVCs and CFFs decreased and there were increased injuries due to being hit by others, sprain, LLFs, SFs, and RSF. With increasing year of admission, the proportion of injuries due to MVCs and mechanical injury decreased. Therefore, we should pay more attention to the injuries due to being hit by others, sprain, and LLFs and consider how to prevent these injuries and treat the LLFs. Male patients presented with a significantly higher proportion of injuries due to low fall, being struck by an object, being hit by others, sprains, ULFs, and a significantly lower proportion of injuries due to MVCs, LLFs, and SFs than female patients. Therefore, we can see that the pattern of TFs among the patients has its own characteristics and that targeted intervention methods should be considered to decrease the incidence and burden of TFs.

In the current study, the incidences of TFs had showed season and month variation, with peaks in summer and September. A significantly lower incidence of TFs was observed in winter than in the other three seasons, which can be explained by less outdoor activity in winter. Fractures are caused by a combination of intrinsic and extrinsic factors that vary with age [5]. The study results are believed to be helpful in the planning and assignment of medical resources for fracture management in children and adolescents [18]. Through the current study, we can see that we should plan many more preventive measures for younger female children, especially injured by MVCs, and assign more medical resources to CFFs in younger children and LLFs in older children.

The most common associated injuries were head and lung injury, so when patients with fracture come to us for treatment, we should pay much attention to the head and lung injury in avoidance of missed diagnosis and delayed diagnosis. The most common complications were fracture malunion and fracture site infection, so effective prevention measures and avoiding the occurrence of such complications are necessary to ensure a safe and successful treatment. This study has several limitations. First, it was limited by the retrospective study design and by the small sample size. Second, there may be selection bias because this study included patients who were referred to our teaching hospitals. Third, the leading causes were low falls, but the exact aetiology such as sport injury of low falls is not mentioned.

## 5. Conclusions

Low falls and upper limb fractures were the leading cause and fracture sites. The epidemiologic features of traumatic fractures have specific annual, age, and gender differences. To further improve the prevention and treatment of traumatic fractures in children and adolescents, policy makers should pay attention to these characteristics.

## Figures and Tables

**Table 1:** The epidemiology of traumatic fractures according to different aetiologies.

Aetiologies	Low fall	MVCs	High fall	Struck by object	Hit by others	Sprain	Mechanical injury	Others	Total
Total	1042	728	350	101	74	49	48	58	2450
Incidence per 100000 HA	111.3	77.8	37.4	10.8	7.9	5.2	5.1	6.2	261.7
M/F	791/251	493/235	248/102	88/13	68/6	43/6	40/8	37/21	1808/642
Mean age	10.8 ± 4.6	11.0 ± 5.3	10.8 ± 5.2	11.2 ± 5.1	14.8 ± 3.2	14.1 ± 3.0	13.6 ± 6.3	12.3 ± 5.3	11.1 ± 5.0
Fracture sites									
ULF	698 (67.0)	125 (17.2)	103 (29.4)	33 (32.7)	31 (41.9)	12 (24.5)	40 (83.3)	26 (44.8)	1068 (43.6)
LLF	237 (22.7)	435 (59.8)	139 (39.7)	42 (41.6)	7 (9.5)	37 (75.5)	5 (10.4)	23 (39.7)	925 (37.8)
CFF	99 (9.5)	225 (30.9)	133 (38.0)	25 (24.8)	40 (54.1)	0	5 (10.4)	11 (19.0)	538 (22.0)
SF	21 (2.0)	41 (5.6)	57 (16.3)	7 (6.9)	0	0	0	4 (6.9)	130 (5.3)
RSF	2 (0.2)	16 (2.2)	8 (2.3)	1 (1.0)	1 (1.4)	0	2 (4.2)	0	30 (1.2)
ND	98 (9.4)	199 (27.3)	130 (37.1)	7 (6.9)	14 (18.9)	3 (6.1)	9 (18.8)	3 (5.2)	463 (18.9)

ULF: upper limb fracture; LLF: lower limb fracture; CFF: craniofacial fracture; SF: spinal fracture; RSF: fracture of rib and sternum; ND: neurological deficit.

**Table 2:** The epidemiology of traumatic fractures according to different year and age range groups.

Groups	Year of admission			Age range group			Total
	2002-2004	2005-2007	2008-2010	≤6	6-12	12-18	
Total	393	893	1164	556	787	1107	2450
Incidence per 100000 HA	186.1	309.7	266.6	59.5	84.1	118.2	261.8
M/F	299/94	660/233	849/315	356/200	577/210 #	875/232 #&	1808/642
Mean age	11.1 ± 4.9	11.0 ± 5.1	11.2 ± 4.9	4.1 ± 1.5	9.4 ± 1.7 #	15.8 ± 1.7#&	11.1 ± 5.0
Aetiologies							
Low fall	155 (39.4)	376 (42.1)	511 (43.9)	219 (39.4)	396 (50.3) #	427 (38.6) &	1042 (42.5)
MVCs	141 (35.9)	266 (29.8) #	321 (27.6) #	203 (36.5)	207 (26.3) #	318 (28.7) #	728 (29.7)
High fall	50 (12.7)	119 (13.3)	181 (15.5)	94 (16.9)	101 (12.8) #	155 (14.0)	350 (14.3)
Struck by object	16 (4.1)	33 (3.7)	52 (4.5)	18 (3.2)	40 (5.1)	43 (3.9)	101 (4.1)
Hit by others	8 (2.0)	40 (4.5) #	26 (2.2) &	1 (0.2)	11 (1.4) #	62 (5.6) #&	74 (3.0)
Sprain	7 (1.8)	14 (1.6)	28 (2.4)	1 (0.2)	15 (1.9) #	33 (3.0) #	49 (2.0)
Mechanical injury	9 (2.3)	28 (3.1)	11 (0.9) #&	10 (1.8)	4 (0.5) #	34 (3.1) &	48 (2.0)
Others	7 (1.8)	17 (1.9)	34 (2.9)	10 (1.8)	13 (1.7)	35 (3.2) &	58 (2.4)
Age range							
≤6/2002-2004	84 (21.4)	213 (23.9)	259 (22.3)	84 (15.1)	141 (17.9)	168 (15.2)	556 (22.7)
6-12/2005-2007	141 (35.9)	285 (31.9)	361 (31.0)	213 (38.3)	285 (36.2)	395 (35.7)	787 (32.1)
12-18/2008-2010	168 (42.7)	395 (44.2)	544 (46.7)	259 (46.6)	361 (45.9)	544 (49.1)	1107 (45.2)
Fracture sites							
ULF	166 (42.2)	401 (44.9)	501 (43.0)	209 (37.6)	403 (51.2) #	456 (41.2) &	1068 (43.6)
LLF	163 (41.5)	307 (34.4) #	455 (39.1) &	198 (35.6)	255 (32.4)	472 (42.6) #&	925 (37.8)
CFF	67 (17.0)	212 (23.7) #	259 (22.3) #	177 (31.8)	167 (21.2) #	194 (17.5) #&	538 (22.0)
SF	25 (6.4)	41 (4.6)	64 (5.5)	5 (0.9)	17 (2.2)	108 (9.8) #&	130 (5.3)
RSF	2 (0.5)	7 (0.8)	21 (1.8)	2 (0.4)	7 (0.9)	21 (1.9) #	30 (1.2)
ND	71 (18.1)	157 (17.6)	235 (20.2)	93 (16.7)	140 (17.8)	230 (20.8) #	463 (18.9)

ULF: upper limb fracture; LLF: lower limb fracture; CFF: craniofacial fracture; SF: spinal fracture; RSF: fracture of rib and sternum; ND: neurological deficit. #: significant difference compared to 2002-2004 year of admission group or ≤6 years old range group; &: significant difference compared to 2005-2007 year of admission group or 6-12 years old range group.

**Table 3:** The epidemiology of TFs according to different genders and whether presented with neurological deficit.

Groups	Genders		Neurological deficit	
	Male	Female	With	Without
Total	1808	642#	463	1987#
Incidence per 100000 HA	193.1	68.6#	49.4	212.2#
Sex ratio	1808/0	0/642#	341/122	1467/520
Mean age	11.5 ± 4.9	10.1 ± 5.1#	11.8 ± 5.1	11.0 ± 4.9#
Age range				
≤6	356 (19.7)	200 (31.2) #	93 (20.1)	463 (23.3)
6-12	577 (31.9)	210 (32.7)	140 (30.2)	647 (32.6)
12-18	875 (48.4)	232 (36.1) #	230 (49.7)	877 (44.1) #
Year range				
2002-2004	299 (16.5)	94 (14.6)	71 (15.3)	322 (16.2)
2005-2007	660 (36.5)	233 (36.3)	157 (33.9)	736 (37.0)
2008-2010	849 (47.0)	315 (49.1)	235 (50.8)	929 (46.8)
Aetiologies				
Low fall	791 (43.8)	251 (39.1) #	98 (21.2)	944 (47.5) #
MVCs	493 (27.3)	235 (36.6) #	199 (43.0)	529 (26.6) #
High fall	248 (13.7)	102 (15.9)	130 (28.1)	220 (11.1) #
Struck by object	88 (4.9)	13 (2.0) #	7 (1.5)	94 (4.7) #
Hit by others	68 (3.8)	6 (0.9) #	14 (3.0)	60 (3.0)
Sprain	43 (2.4)	6 (0.9) #	3 (0.6)	46 (2.3) #
Mechanical injury	40 (2.2)	8 (1.2)	9 (1.9)	39 (2.0)
Others	37 (2.0)	21 (3.3)	3 (0.6)	55 (2.8) #
Fracture sites	1808	642		
ULF	841 (46.5)	227 (35.4) #	146 (31.5)	922 (46.4) #
LLF	650 (36.0)	275 (42.8) #	126 (27.2)	799 (40.2) #
CFF	382 (21.1)	156 (24.3)	231 (49.9)	307 (15.5) #
SF	82 (4.5)	48 (7.5) #	63 (13.6)	67 (3.4) #
RSF	18 (1.0)	12 (1.9)	13 (2.8)	17 (0.9) #
ND	341 (18.9)	122 (19.0)	463 (100.0)	0 #

ULF: upper limb fracture; LLF: lower limb fracture; CFF: craniofacial fracture; SF: spinal fracture; RSF: fracture of rib and sternum; ND: neurological deficit.

#: significant difference compared to male patients or to patients with neurological deficits

## Data Availability

The data used to support the findings of this study are included within the article.
